# Renal Cell Carcinoma Metastasizing to Left Atrium With Coronary Sinus Invasion: A Rare Site of Metastasis Mimicking Myxoma

**DOI:** 10.3389/fonc.2019.00738

**Published:** 2019-08-07

**Authors:** Gerard Nkengurutse, Qi Wang, Feng Tian, Sixiong Jiang, Liang Zhang, Weibing Sun

**Affiliations:** Department of Urology, The Second Affiliated Hospital of Dalian Medical University, Dalian, China

**Keywords:** renal cell carcinoma, RCC, left atrium, coronary sinus, metastasis, myxoma

## Abstract

Renal cell carcinoma (RCC) metastasizing to the heart with inferior vena cava (IVC) involvement is well-documented. However, its metastasis to the right heart without venous involvement is very rare. To the left atrium, metastasis is even rarer with only a few cases reported in medical literature. Herein, we report a case of a 56-year-old man who presented to our department for the treatment of a right renal mass and a right adrenal mass discovered on a follow-up plain computed tomography (CT) 13 years after left laparoscopic radical nephrectomy. During the workup, a transthoracic echocardiography (TTE) revealed a left atrial mass with a suspicion of a myxoma. This finding prompted a cardiac surgery consult which proposed a surgical removal of the mass. Intraoperatively, the tumor was found to invade the coronary sinus as well. The entire tumor was successfully removed and surgical repair of the unroofed coronary sinus was performed. Pathological examination of the tumor along with immunohistochemistry–showing positivity for CAIX, CD10, Vimentin, and PAX-8–pointed to a diagnosis of metastatic clear cell RCC. Eight months postoperatively, he was free of any symptom. In conclusion, RCC metastasizing to the left atrium is extremely rare. A comprehensive search revealed only nine reports in the literature. We report, to our knowledge, the first case of RCC metastasizing to the left atrium with concomitant invasion of coronary sinus. Surgical resection combined with unroofed coronary sinus repair allowed a complete removal of the tumor. In patients with a history of RCC, a metastasis should be thought of when a left atrial mass is present.

## Introduction

Renal cell carcinoma (RCC) is an aggressive malignancy representing 2–3% of all cancers, and it is the most lethal of all urologic cancers. In 2018, there were approximately 136,500 new cases of renal cancer and 54,700 kidney-cancer-related deaths in Europe (1).

RCC is a highly metastatic malignancy with 20–30% of patients having a metastatic or a locally advanced disease at the time of diagnosis (2). While metastasis can occur almost anywhere, the most common sites of metastasis are the lungs, the liver, bones and the brain.

Cardiac metastases from RCC are uncommon. In most cases, cardiac metastases are concomitant with IVC tumor thrombus where a further extension reaching the right atrium is found in approximately 1% of all patients (3). However, metastasis to the right heart without IVC involvement is very rare. To the left atrium, metastasis is even rarer with only a few reports in the literature.

Herein, we report a rare case of left atrial metastasis with coronary sinus invasion 13 years after left laparoscopic radical nephrectomy for RCC and provide a review of the relevant literature.

## Case Presentation

A 56-year-old Chinese male presented to our department in September 2018 with a right renal mass and a right adrenal mass discovered on a follow-up plain CT half a month before. The patient had left radical nephrectomy 13 years before for left RCC and he had a regular follow-up without any sign of recurrence or metastasis. Physical examination was unremarkable and the patient was otherwise doing well without any symptom. He had type 2 diabetes mellitus for 9 years wherein metformin and insulin were used to control blood glucose. A radical operation for rectal cancer was performed 7 years before. He had a history of heavy cigarette smoking with an average of 20 cigarettes per day for 60 years and a 60-year history of alcohol drinking (average 1 bottle of beer per day).

**Figure 1:**
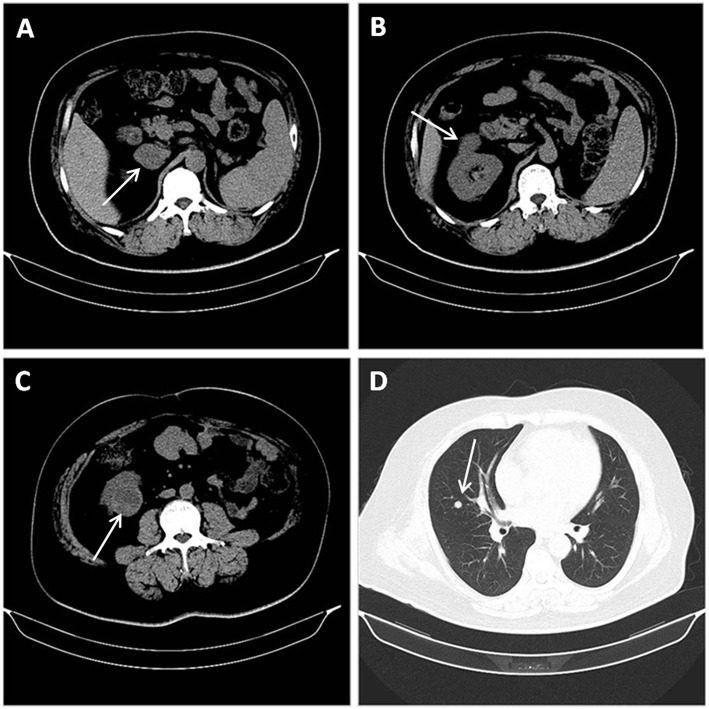
Abdominal and pelvic non-contrast CT revealing a 36 × 31-mm, well-demarcated, ellipsoid and hypodense mass with a CT value of about 27 Hounsfield units (HU) in the external branch of the right adrenal region. **(A)** Hypodense lesions with a diameter of 15 mm and 39 mm discovered in the upper **(B)**, and lower **(C)** poles of the right kidney, respectively. Non-contract CT of the chest suggesting lung metastasis: multiple nodules in both lungs, the largest one being located in middle lobe of the right lung **(D)**.

Work-up: An abdominal and pelvic non-contrast CT revealed a 36 × 31-mm, ellipsoid, and hypodense mass in the external branch of the right adrenal region (Figure 1A). It was well-demarcated with a CT value of about 27 Hounsfield units (HU). Hypodense lesions with a diameter of 15 mm and 39 mm were discovered in the upper and lower poles of the right kidney, respectively (Figures 1B,C). Their mean CT attenuation value was 39 HU. No renal vein or IVC tumor thrombus was visualized. Routine blood tests (including LDH, calcium) were unremarkable.

**Figure 2:**
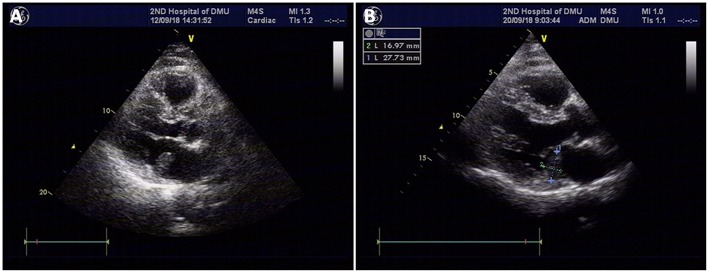
TTE revealing a 23.9 × 13.4-mm, hyperechoic mass with a smooth surface in the left atrium, close to the posterior leaflet of the mitral valve, and moving without extension to the outflow tract during the cardiac cycle; the mass was suspected to be a myxoma **(A)**. Repeat TTE showing a 27.7 × 16-mm isoechoic mass attached to the posterior leaflet annulus of the mitral valve in the enlarged left atrium **(B)**.

During the patient evaluation phase, a non-contract CT of the chest revealed multiple nodules in both lungs, the largest one being located in the middle lobe of the right lung (Figure 1D). Compared with findings of a follow-up chest CT taken in February 2016, some of the nodules were enlarged, suggesting a metastasis. No abnormality was found on the heart and big vessels; and no mass or enlarged lymph nodes were seen in the mediastinum. A TTE was performed, revealing a 23.9 × 13.4-mm, hyperechoic mass with a smooth surface in the left atrium, close to the posterior leaflet of the mitral valve, and moving without extension to the outflow tract during the cardiac cycle (Figure 2A). This imaging modality suspected a diagnosis of myxoma. The ejection fraction was 62% and no abnormality was found on electrocardiography.

Prior to any other intervention, a cardiac surgery consult was sought, suggesting surgical removal of the mass due to the risk of it to detach. The patient was then transferred to cardiac surgery department where a repeat TTE showed a 27.7 × 16-mm isoechoic mass attached to the posterior leaflet annulus of the mitral valve in the enlarged left atrium (Figure 2B). While the mass was following the mitral annulus moving toward the left atrial side during early diastole, it was tending to move toward the mitral valve orifice during late diastole and systole. Transesophageal echocardiography (TEE) revealed an enlarged left atrium and a 27.7 × 16-mm, isoechoic atrial mass of inhomogeneous echogenicity. It was attached with a broad base between the posterior leaflet of the mitral valve and the coronary sinus. A part of the atrial wall was protruded outwards and there was no clear margin between the protrusion and the coronary sinus. A capsule could be visualized around the mass. Despite the metastatic status of the patient, surgical removal of the atrial mass was scheduled since there was a risk of it to detach and cause embolism.

**Figure 3:**
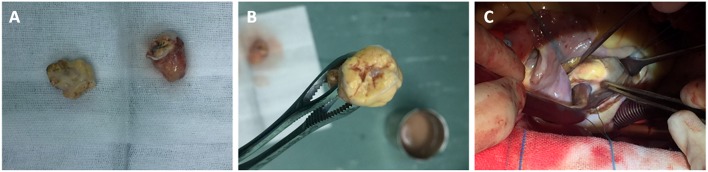
Two specimens of 2.7 × 1.5 × 1.5 cm, and 2 × 1.7 × 0.7 cm **(A)**. They were yellowish and cystic-solid with a tumor pseudocapsule **(B,C)**.

**Figure 4:**
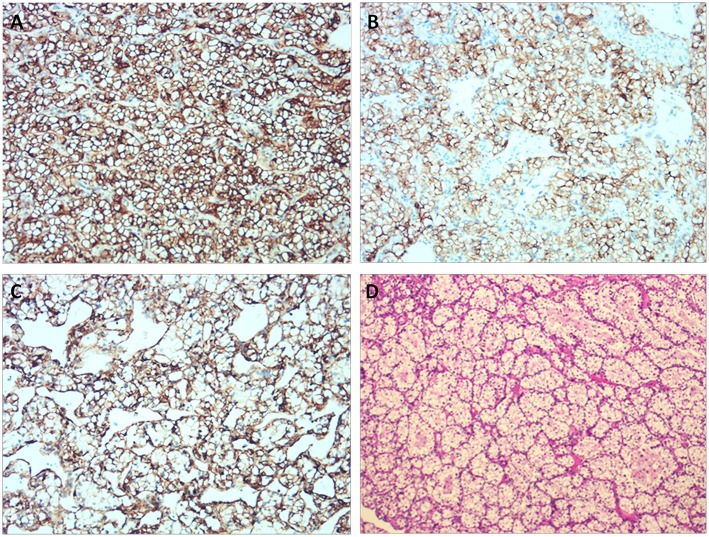
Immunohistochemical staining showing positivity for CAIX, CD10, and Vimentin. **(A)** CAIX (×200). **(B)** CD10 (×200). **(C)** Vimentin (×200). **(D)** Histological examination of the tumor using Hematoxylin & Eosin stain.

Through a median sternotomy, cardiopulmonary bypass (CPB) was established by cannulating the ascending aorta and the superior and inferior vena cava. The plan was a hypothermic CPB with cold cardioplegia. After and through right atriotomy, an incision was made in the fossa ovalis, exposing a yellow mass close to the posterior leaflet of the mitral valve. It was a mass with a broad base rather than a pedicle. When the part of the tumor protruding inwards was entirely removed, the remaining part of the tumor was found to be in immediate vicinity of the coronary sinus. Via a longitudinal incision in coronary sinus, the tumor was entirely removed with its intact pseudocapsule. An exploration of the left atrium and the opening of pulmonary veins did not reveal any residual tumor. An artificial pericardial patch was applied to the incised part of the coronary sinus and the latter was successfully repaired without causing any leakage. Pericardial patch was also used to close the fossa ovalis and the right atriotomy was closed. Post-operative TEE did not reveal any residual tumor or abnormality in coronary sinus drainage. Two specimens of 2.7 × 1.5 × 1.5 cm and 2 × 1.7 × 0.7 cm were obtained (Figure 3A). They were yellowish and cystic-solid with a tumor pseudocapsule (Figures 3B,C). Microscopically, the tumor cells were of clear cytoplasm with an acinar architecture (Figure 4D). Immunohistochemical staining showed positivity for CAIX, CD10,Vimentin, and PAX-8 and negativity for CK7, calretinin, WT1, WT1,CK5/6, CK20, Villin, and CDX2 (Figure 4). The postoperative period was uneventful. The patient was discharged with a recommendation to consult the department of cardiac surgery after 1, 3, and 6 months, and the departments of urology and oncology to ensure a multidisciplinary treatment. Four months postoperatively (on 2019 January 31st), he was contacted by telephone and he reported not seeking any further treatment because he was free of any symptom. We contacted the patient for the second time after 8 months (on 2019 May 27th). He was doing well and was going to his daily activities without any symptom. He denied any targeted therapy or immunotherapy use. We recommended him again to recheck and seek management for the renal and adrenal masses.

## Discussion

RCC is an aggressive disease accounting for 3% of all human malignancies and it is the most lethal of all the urologic cancers. In 2018, there were ~136,500 new cases of renal cancer and 54,700 kidney-cancer-related deaths in Europe (1). Its incidence has been increasing due in part to lifestyle factors and the widespread use of imaging studies, allowing many RCC to be discovered incidentally. Indeed, the classical presentation of RCC—the triad of gross hematuria, flank pain and palpable abdominal mass—is nowadays not common. This triad has been recently sometimes described as the “too late triad.”

RCC is characterized by a high propensity to metastasis. On presentation, 25% of patients already have a metastatic or a locally advanced disease. The most common sites of metastasis are the lungs, the liver, bones, the brain and the adrenal glands. However, metastatic RCC to the heart are very rare, most of which occurring concomitantly with IVC involvement. Without IVC involvement, cardiac metastases in general and left atrial metastases in particular, are even rarer, with only a few cases reported in the literature. Herein, we present a case of LA metastasis from RCC in a patient who had undergone left laparoscopic radical nephrectomy 13 years before. We further reviewed previously published cases and summarized the findings in Table 1.

**Table 1:** Patients' characteristics and the course of disease in all reported LA metastases from RCC.

**References**	**Age(years)/Sex**	**Presentation**	**Other sites of metastasis**	**Treatment**	**Years from nephrectomy**	**Diagnostic tools**	**IHC**	**Follow-up (months) and outcome**
Patane et al. (4)	58/F	asymptomatic	Left lower lobe of the lung, PV	Resection of the LA mass through sternotomy, video-assisted left lower lobectomy	ND	ND	ND	No recurrence, 13 months
Fogel et al. (5)	77/M	dizziness, syncope, dyspnea, atrial fibrillation	Left lung, PV	Left pneumonectomy, partial atrectomy through left thoracotomy	8	History, TTE, MRI, intraoperative findings	ND	Died postoperatively of respiratory complications
Miyamoto et al. (6)	56/M	Syncope	Mediastinal LN, right inferior PV, previously in lungs, intra-abdominal, spine.	Resection of the LA mass through sternotomy, lymphadenectomy	3	History, chest CT, TEE, histopathology	ND	No recurrence, 4 months
Cochennec et al. (7)	42/F	asymptomatic	Left lower lobe of the lung, left inferior PV	Resection of LA mass and of the left lower lobe though sternotomy associated with left anterolateral thoracotomy	4 and 2 from left and right nephrectomies respectively	History, chest CT, TEE, PET, histopathology, IHC	(+): cytokeratin AE1/AE3, vimentin and CD10. (–): CK 7 and CK20.	No recurrence, 8 months
Seker et al. (8)	37/M	Numbness, cerebellar and thalamic acute ischemic lesions	Left lung, muscle, cerebellar tentorium, left inferior PV	Tyrosine kinase inhibitor (axitinib)+ anticoagulant	6	History, PET, TEE, cardiac MRI, coronary CT,	ND	ND
Tolay et al. (9)	56/F	dyspnea	Right hilar LN, right PV, previously in both lungs	mTOR inhibitor (Temsirolimus)	3	History, chest CT	ND	11 months, stable disease
Tabakci et al. (10)	48/F	cough and hemoptysis	Left lower lobe of lung, left inferior PV	Tyrosine kinase inhibitor (sunitinib)	8	Chest CT, PET, lung and LN biopsy, IHC	CD10 (+)	ND
Ohba et al. (11)	75/M	consciousness disturbance	Both lungs, lymph nodes, right superior PV, pubic bone	Complete surgical resection of the LA mass, Interferon-alpha, sorafenib then everolimus for lung metastasis, radiotherapy for pubic bone metastasis	4	History, TTE, histopathology	ND	Died from progressive disease 4 months postoperatively.
Strauch et al. (12)	51/F	Dyspnea, hypertension, atrial fibrillation	Right lower PV	Tumor removal through left atriotomy+ Tyrosine kinase inhibitor (sunitinib)	1/4	History, chest CT,MRI, histopathology	pancytokeratin-expressing areas of the previously resected RCC	Continued to do well at 6 months with unobtrusive chest CT
Present case	56/M	asymptomatic	Both lungs, coronary sinus, right kidney, right adrenal gland	Resection of the atrial mass through sternotomy, Surgical repair of the unroofed coronary sinus	13	History, TTE, TEE, histopathology, IHC	(+): CAIX, CD10, vimentin and PAX-8. (–): CK7,calretinin,WT1, WT1,CK5/6, CK20, Villin and CDX2	No recurrence, Free of symptoms, 8 months

*LA, left atrium; IHC, Immunohistochemistry; PV, pulmonary vein; NS, Not stated; CT, computed tomography; MRI, Magnetic resonance imaging; PET, Positron emission tomography; LN, lymph node; TTE, Transthoracic echocardiography; TEE, Transesophageal echocardiography; ND, No data available*.

A comprehensive search in PubMed and Web of Science was conducted using keywords and Boolean operators as follows: [(metastasis OR metastatic) AND (renal cell carcinoma OR renal cancer)] AND (atrial OR atrium). The search was updated till February 24th, 2019 without any language restriction to detect LA metastases from RCC without IVC involvement. A hand search in Google scholar was also performed. To date, only 9 cases are available in the literature (4–12). The present report is the 10th case of LA metastasis from RCC. We did not find any previous report describing LA metastasis with concomitant coronary sinus invasion.

Due to the risk of sudden death, embolism, and intraoperative or perioperative events in every patient with an intracardiac mass (11), it is wise to seek a cardiovascular or cardiothoracic surgery consult in such patients before any other intervention. Myxoma will be suspected first as it is the most common primary cardiac tumor (5). In fact, in the biopsy of 266 patients with cardiac masses, only 1.1% were RCC (13). In our patient, imaging study (TTE) pointed to a myxoma (see Figure 2), prompting a cardiac surgery consult that suggested a surgical removal of the mass. During the operation, the tumor was found to invade the coronary sinus and a longitudinal incision in the coronary sinus allowed an entire removal of the tumor with its intact pseudocapsule. Unroofed coronary sinus repair was then performed. A postoperative TEE did not reveal any mass. Immunohistochemical staining pointed to RCC and the diagnosis was confirmed given the patient's history.

Cardiac metastases from RCC are thought to occur via two mechanisms: The venous hematogenous spread through renal vein to the right heart and the lymphatic spread via carinal lymph nodes and parasternal lymph vessels. It has been reported that hematogenous spread is the most commonly involved pathway in metastasis to the right heart while the lymphatic spread is frequently involved in metastasis to the left heart (11). Left heart metastasis from RCC is frequently associated with metastasis to other sites. The present case is in line with this pattern because the patient was hospitalized for the management of right renal and right adrenal masses which were thought to be metastases. Moreover, pulmonary metastasis was considered since chest CT revealed masses that increased in size compared to previous CTs of 2016.

RCC has a characteristic feature of a high propensity to venous invasion. On review of literature, except the present case, all the previously reported cases of LA metastasis from RCC revealed pulmonary veins involvement. Apart from the report by Miyamoto et al. (6) where a direct invasion of pulmonary vein from a mediastinal lymph node was confirmed, most reports mentioned the lung as the source of pulmonary vein, and LA metastasis (4, 5, 7, 8), or could not rule out such source (9). While previous reports may apparently suggest a hypothesis that all LA metastases from RCC occur via pulmonary veins (although the small sample size cannot allow to draw further conclusions), the coronary sinus invasion in the present case and the absence of pulmonary vein tumor thrombus may raise a suspicion of another pathway in which LA metastasis can occur via coronary sinus—coronary sinus tumor thrombus invading the LA. However, intraoperative findings could not clearly support such a pathway. Moreover, the presence of lung metastasis and the absence of any mass or enlarged lymph nodes in mediastinum in the present case favor the pulmonary vein as the route of metastasis to left atrium. A diagnosis of lung metastasis from RCC—either concurrently or previously—was present in most cases (8 out of 9). Moreover, no isolated LA metastasis from RCC has been reported thus far. The time from nephrectomy to LA metastasis ranged from 3 months to 13 years, our present case having the longest time. There are no specific signs and symptoms for LA metastasis from RCC. Patients may be asymptomatic (3 out of 9) or may present with non-specific symptoms like dyspnea (3 out of 9), syncope (2 out of 9), consciousness disturbance or cough.

There is no established standard management for cardiac metastases from RCC. That said, metastasectomy is still playing an important role in RCC treatment and it has been shown to improve both overall survival and cancer-specific survival (14). Metastasectomies either synchronous or metachronous with nephrectomies are described especially in the liver and lungs in selected patients. Given the paucity of data regarding treatment of such cardiac metastases, management of RCC with cavoatrial extension—where radical surgical intervention is the only option to potentially result in curative treatment—may constitute a relatively better source of data. Due to the fear of cardiac sudden death, most cardiac masses are removed as soon as possible. Most of the cardiac metastases from RCC reported thus far were treated with surgical removal (4–7, 11, 12). Cardiopulmonary bypass (CPB) with or without hypothermia—usually performed through a sternotomy approach—is the most commonly used surgical technique. While a number of surgical strategies for the management of RCC with cavoatrial extension are available without a clear evidence of superiority of one technique over the others, Gaudino, and associates (15) reported in their systematic review that CPB with Deep Hypothermic Circulatory Arrest (DHCA) is the more commonly used technique when the level of invasion is above the diaphragm and it has the advantages of possible organ preservation and an excellent exposure with bloodless operative field, thus aiding in complete resection. However, in addition to its disadvantages—longer operative time, coagulopathy, complexity—the common belief that CPB with DHCA increases the operative risk and major complication rate (though not proven by the Gaudino and associates) may be a factor for surgeons to prefer CPB without DHCA. It is worth noting that these techniques' safety and treatment outcomes were primarily studied on RCC extending to IVC and right heart and thus will not necessarily yield the same results when applied on left atrial metastases without IVC involvement. In our case, successful extirpation of the tumor from the coronary sinus was followed by repair of the unroofed coronary sinus. When cardiac metastases are deemed inoperable, conservative approach with molecular targeted therapy is a treatment option although it has been rarely reported. Tyrosine kinase inhibitors (especially sunitinib and pazopanib) are reported to result in partial response or stable disease in such setting (16, 17). Mammalian target of rapamycin (mTOR) inhibitors were also reported with a stable disease as the main outcome (9, 18). The well-documented report by Tolay et al. (9) describes significant regression of cardiac metastasis from RCC and improvement of symptoms after 14 weeks of mTOR inhibitor therapy.

Currently, there is limited evidence regarding the efficacy and safety of immune checkpoint inhibitors in patients with intracardiac metastases. To date, only one report by Ansari et al. (19) described an excellent response to nivolumab (a programmed death-1 (PD-1) receptor inhibitor) where 12 months of treatment with this drug resulted in 70% reduction in the size of the intracardiac RCC metastasis on enhanced chest CT. However, no studies have compared long-term outcomes of these different treatment modalities in this subpopulation.

In conclusion, RCC metastasizing to the left atrium is extremely rare with only 9 reports in the literature. We report the 10th case of LA metastasis of RCC occurring 13 years post radical nephrectomy. This case highlights the need for a metastatic RCC to be considered when a left atrial mass is present in a patient with a history of renal cancer. To our knowledge, this is the first case of RCC metastasizing to the left atrium with concomitant invasion of coronary sinus. Surgical resection combined with unroofed coronary sinus repair allowed a successful removal of the entire tumor. In the absence of standard therapy for such cardiac metastasis, surgical removal appears to be both a diagnostic and therapeutic tool, preventing the risk of heart failure and sudden death. In surgically unresectable cases, conservative therapy with molecular targeted therapy (either tyrosine kinase inhibitors or mTOR inhibitors) constitutes a treatment option. Despite the limited evidence regarding the efficacy and safety of immune checkpoint inhibitors for patients with intracardiac RCC metastases, these agents may improve outcomes in this subpopulation.

## Data Availability

All datasets generated for this study are included in the manuscript/supplementary files.

## Informed Consent

A written informed consent to publish the report and associated medical images was obtained from the patient.

## Author Contributions

GN and QW collected and analyzed the patient's clinical data and designed the research. GN performed the review of literature and drafted the manuscript. QW, FT, SJ, WS, and LZ revised the manuscript. WS and SJ supervised the report and the publication process. All authors have read and approved the final version of the manuscript.

### Conflict of Interest Statement

The authors declare that the research was conducted in the absence of any commercial or financial relationships that could be construed as a potential conflict of interest.
